# Matrix metalloproteinase‐9 regulates cell adhesion and membrane protrusive activity of ovarian cancer cells

**DOI:** 10.1002/2211-5463.70220

**Published:** 2026-02-24

**Authors:** Claire Strauel, Sam Bennett, Natalie Ehlinger, Nelson Cook, Katelyn Marvin, Caleb L. Lines, Sam Reyes, Brooke Kirby, Shawn M. Ellerbroek

**Affiliations:** ^1^ Department of BCES Wartburg College Waverly Lowa USA

**Keywords:** adhesion, E‐cadherin, EGF, MMP9, Ovarian, protrusion

## Abstract

Matrix metalloproteinase‐9 (MMP9), an extracellular endopeptidase, is upregulated by Epidermal Growth Factor (EGF) signaling and associated with ovarian epithelial cancer progression. To further understand the contribution of MMP9 to EGF‐stimulated metastatic behavior, we created MMP9‐null cells (M9‐KO) from an ovarian cancer cell line. This model showed MMP9 loss did not block EGF‐driven E‐cadherin dissolution and associated epithelial‐to‐mesenchymal transition. However, MMP9‐null cells exhibited delayed and reduced EGF‐driven actin‐based membrane protrusions, with transient re‐expression of MMP9 sufficient to drive both lamellipodial and filopodial membrane protrusions. Finally, M9‐KO cells exhibited a reduction of adhesion to extracellular matrices, which was significantly recovered by stable re‐expression of MMP9. Together, these results provide direct evidence that MMP9 mediates EGF‐driven membrane protrusions and promotes ovarian cancer cell adhesion to mesothelial extracellular matrices.

**Impact statement**

This work provides direct evidence that MMP9 mediates EGF‐driven membrane protrusions and promotes ovarian cancer cell adhesion to mesothelial extracellular matrices.

## Graphical abstract

Matrix metalloproteinase‐9 (MMP9) drives ovarian cancer progression. Using MMP9‐null cells (M9‐KO) created from ovarian cancer cells, we found MMP9 loss did not block Epidermal Growth Factor (EGF)‐driven E‐cadherin dissolution or EMT but delayed and reduced EGF‐driven membrane protrusions. Transient MMP9 re‐expression drove membrane protrusion. M9‐KO cells exhibited 50–60% reduction in extracellular matrix adhesion. Stable re‐expression of MMP9 significantly recovered adhesion.

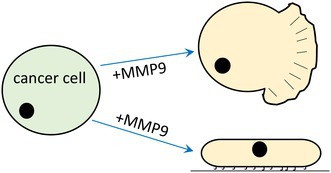

**Abbreviations**
EGF: epidermal growth factor
EMT: epithelial to mesenchymal transition
MMP9: matrix metalloproteinase‐9
OEC: ovarian epithelial cancer
TIMP1: tissue inhibitor of matrix metalloproteinase‐1

Ovarian epithelial cancer (OEC) is the most lethal gynecologic malignancy with a survival rate of less than 50% [1, 2]. The expression of matrix metalloproteinase‐9 (MMP9), an extracellular zinc‐dependent proteinase, positively correlates with OEC progression [3, 4]. MMP9 binds to several proteins and boasts a range of substrates, resulting in multifaceted contributions to OEC progression [5, 6]. MMP9 is considered a promising anticancer therapeutic target; however, off‐target toxicity and lack of catalytic inhibitor selectivity have been impediments [5]. A better understanding of how MMP9 promotes cancer progression will help refine and focus targeted therapy.

Two extracellular targets of MMP9‐mediated hydrolysis are the proteins E‐cadherin and Claudin‐5, whose cleavage is hypothesized to advance cell–cell junction dissolution and OEC spheroid release from a primary tumor site [1, 7, 8]. Once released into the peritoneal fluid, OEC cells can undergo transcoelomic metastasis by binding to the collagen, fibronectin, and laminin‐rich mesothelial layer of the peritoneum [9, 10, 11]. OEC cellular engagement of these matrices is principally through β1‐integrin heterodimers and CD44 receptors [12, 13]. MMP9 has been reported to directly bind these receptors [14, 15], opening the door for regulatory consequences. For example, MMP9 binding to CD44 has been reported to facilitate focalized cleavage of latent TGF‐β [16]. At the same time, binding of MMP9's hemopexin domain to β1‐integrin heterodimers is sufficient to elicit a cell signaling cascade that strengthens a survival phenotype in B‐cell chronic lymphocytic leukemia cells [17]. Metastatic growth of OEC cells into a lesion within the mesothelial layer is hypothesized to require MMP9 modification of the tumor microenvironment, including cleavage of matrix proteins, cytokines, chemokines, and growth factors [5, 6]. The sum impact of this MMP9 activity is postulated to include increased cell growth, motility, attenuation of the body's antitumor immune response, and stimulation of pro‐angiogenic signals [18].

One of the earliest clues regarding regulation of OEC expression of MMP9 was the observation that primary ovarian cancer cells quickly lose MMP9 expression when passaged in cell culture [19]. This suggested factor(s) in the tumor microenvironment play a large role in driving and regulating MMP9 expression *in situ* [20]. One likely regulator is Epidermal Growth Factor (EGF), which is both elevated in patients' ascites fluid and an established driver of MMP9 expression in a number of established OEC cell lines [21, 22]. The EGF‐receptor (EGFR) is overexpressed on the surface of malignant OEC cells and correlates with poor patient prognosis [23, 24]. Additionally, its signaling elicits phenotypic changes in most OEC cell lines that resemble epithelial–mesenchymal transition (EMT) [24].

As MMP9 is implicated in a number of prometastatic processes and upregulated by EGFR activation, we hypothesized that loss of MMP9 would negatively impact EGF stimulation of ovarian cancer cell EMT and activity. We report here that loss of MMP9 does not block EGF‐driven E‐cadherin dissolution and associated EMT phenotype, but instead delays and reduces EGF‐driven membrane protrusions. Further, loss of MMP9 reduced OEC cell adhesion to matrices common in mesothelium, and re‐expression of MMP9 significantly restored adhesion.

## Materials and methods

### Cell culture and cell lines

OVCA 433 (RRID:CVCL_0475), an ovarian serous adenocarcinoma cell line, was provided by Dr. Sharon Stack (Harper Cancer Research Institute, South Bend, IN). Cell cultures were maintained on 10‐cm dishes in the presence of minimum essential medium (Invitrogen, Carlsbad, CA, USA) supplemented with 10% fetal bovine serum (Biowhittaker, Walkersville, MD, USA).

To create an MMP‐9 null OVCA433 cell line, cells were cotransfected with a plasmid containing a puromycin resistance gene flanked by LHA and RHA MMP9 recombination sequences and a pCas9 guide plasmid coding for either the genomic guide sequence TCCACCCTTGTGCTCTTCCC or GGTCTCCAGGGAAGAGCACA (Origene, Rockville, MD, USA). All transfections were completed with MegaTran transfection reagent (Origene), a nonlipid PEI cationic polymer system, according to the manufacturer's protocol. Puromycin (Gibco, Waltham, MA, USA) resistant clones were isolated and screened for the absence of MMP9 activity by gelatin zymography and western blotting. The genome of each isolated MMP9‐null was subsequently sequenced (Elim Biopharmaceuticals, Hayward, CA, USA) to confirm biallelic disruption of the MMP9 gene. Seven null clones were pooled to establish a new cell line, M9‐KO. To create a re‐expression cell line, first passage M9‐KO cells were transfected with pCDNA3.1‐MMP9 (a gift of Dr. Rafi Fridman, Wayne State University). After hygromycin (Sigma Aldrich, St. Louis, MO, USA) selection, five unique stable clones were isolated and confirmed to re‐express MMP9 through gelatin zymography analysis. All five clones were pooled to create the new cell line, M9‐KO + MMP9. All experiments were performed with mycoplasma‐free cells. All three human cell lines used in this study were authenticated using STR profiling prior to submission for publication. STR profiling was completed by American Type Culture Collection (ATCC) using an ABI Prism 3500xl Genetic Analyzer (Barcodes STRD3303, STRD3304, STRD0851).

### Gelatin zymography

MMP‐9 activity in conditioned media was determined using SDS/polyacrylamide gel electrophoresis zymography. The conditioned media from cells treated under serum‐free conditions were collected and diluted with 5X nonreducing Laemmli buffer before loading onto 9% SDS/PAGE gels containing 0.1% gelatin [25]. SDS was removed through a 1‐h incubation in 2.5% Triton‐X‐100, and gels were incubated in 20 mm glycine, 10 mm CaCl_2_, 1 μm ZnCl_2_ (pH 8.3), at 37 °C for 24–36 h prior to staining for gelatin with Coomassie Blue. Enzyme activity was visualized as zones of gelatin clearance within the gels.

### Western blotting

Whole cell lysates (scrape collected in 1X‐Laemmli Buffer) or serum‐free conditioned media were resolved on 4–20% gradient polyacrylamide Tris‐Glycine gels (Invitrogen). All samples were clarified at 15000 × ***g*** and reduced before analysis. Resolved proteins were transferred onto PVDF membrane (Millipore, Burlington, MA, USA), blocked in 5% milk in TBST (25 mM Tris–HCl, pH 7.4, 150 mm NaCl, 0.1% Tween‐20), and probed using either anti‐MMP9 mouse monoclonal antibody (GE‐213; Invitrogen), anti‐β1‐integrin polyclonal antibody (PA5‐78028; Invitrogen), anti‐E‐cadherin monoclonal antibody (HECD‐1; Invitrogen), anti‐CD44v6 monoclonal antibody (Clone VFF‐18; Invitrogen), or anti‐Beta‐Actin monoclonal antibody (BA3R; Invitrogen). Results were visualized using HRP‐conjugated goat anti‐mouse or anti‐rabbit antibody (ImmunoResearch Laboratories, West Grove, PA, USA), enhanced chemiluminescence reagent (Pierce, Rockford, IL, USA), and an iBrightCL1000 (Invitrogen).

### Reverse transcription quantitative PCR

OVCA mRNA was isolated using TriZol reagent (Invitrogen). For analysis of EGF‐stimulated cells, cells were washed twice in serum‐free media and then incubated for 24 h in control serum‐free media or serum‐free media supplemented with 100 nm EGF (PHG0311; Gibco). Isolated mRNA was converted to cDNA using a TaqMan Reverse Transcriptase Kit (Applied Biosystems). Transcript levels were quantified using TaqMan Real‐Time PCR master mix (Applied Biosystems) and a StepOnePlus Real‐Time PCR system. All values were normalized to actin transcript levels. Primers used were Hs01124232_G1 (Cortactin/CTTN), Hs01023895_G1 (ECAD/CDH1), Hs99999903_m1 (Actin), Hs00958880 (uPAR), Hs01092512_G1 (TIMP1), Hs00901885_G1 (EpCAM), Hs00957562_G1 (MMP9), and Hs00559840_G1 (KRT7) from Applied Biosystems.

### Fluorescence microscopy

For E‐cadherin immunofluorescence, cells were plated on glass‐coverslips overnight at 40% confluency, treated as indicated, fixed for 10 min in 3.7% formaldehyde/phosphate buffered saline (PBS), and then permeabilized for 5 min with 0.5% TX‐100/PBS. PBS‐washed cells were subsequently incubated with 1 μg·mL^−1^ anti‐E‐Cadherin mAb (HECD‐1; Invitrogen) for one hour at 37 °C. Staining was visualized using an Alexa Fluor 488 goat anti‐mouse secondary antibody (Jackson ImmunoResearch, West Grove, PA, USA). For MMP9 immunofluorescence, cells were plated on glass‐coverslips overnight and then washed and fixed for 10 min in 3.7% formaldehyde/phosphate buffered saline (PBS). Washed nonpermeabilized cells were incubated with 1 μg·mL^−1^ anti‐MMP9 mAb (GE‐213; Invitrogen) for one hour at 37 °C. Staining was visualized using an Alexa Fluor 488 goat anti‐mouse secondary antibody (Jackson ImmunoResearch).

For experiments involving transfected cells, cells were plated overnight on glass coverslip in six‐well plates before being transfected with the indicated plasmids (range 150–800 ng). Approximately 6 h post transfection, cells were washed twice with excess serum‐free media and incubated 18–24 h before fixation as described above. For filamentous‐actin staining, coverslip‐adherent cells were transfected with pcDNA3.1‐MMP‐9, incubated for 16 h, fixed for 10 min in 3.7% formaldehyde/PBS, permeabilized for 5 min with 0.1% TX‐100/PBS, PBS‐washed, and then incubated with Rhodamine‐Phalloidin (Invitrogen). For β1‐integrin staining, coverslip‐adherent cells were fixed for 10 min in 3.7% formaldehyde/PBS, permeabilized for 5 min with 0.1% TX‐100/PBS, PBS‐washed, and blocked with 2% BSA (Sigma Aldrich) for 45 min at room temperature. Cells were then incubated with 15 μg·mL^−1^ anti‐β1‐integrin polyclonal antibody (PA5‐78028, Invitrogen) for one hour at 37 °C. Staining was visualized using a Texas‐Red goat anti‐rabbit secondary antibody (Jackson ImmunoResearch) containing NucBlue ReadyProbes DAPI reagent (Molecular Probes, Carlsbad, CA, USA). All fluorescence images of washed and mounted (PBS/glycerol) cells were obtained on an Olympus spinning disk confocal microscope using a CoolSNAP E2 CCD camera (Photometrics, Tucson, AZ, USA) and Nikon Image software.

### Membrane protrusion analysis

Cells were seeded on glass coverslips in serum media (six‐well plate) and maintained for 36–48 h before reaching a target confluence of 30–40%. For experiments involving transfected cells, the indicated cell line was grown overnight to 30–40% confluency on glass coverslips prior to transfection (Megatran, Rockville, MD, USA) with either 150 ng pCMV‐Myc control plasmid or pCDNA3.1‐MMP9 and a 24‐h expression period in serum‐containing media. Prior to analysis, all coverslip‐adherent cells were washed twice with 2 mL of serum‐free media and then treated with serum‐free media in the absence or presence of 100 ng EGF. Some serum‐free media also contained 0.1% DMSO vehicle or the MMP inhibitor GM‐6001 (Millipore). After the indicated time, coverslip‐adherent cells were immediately fixed in 3.7% formaldehyde/PBS, washed, and mounted (PBS/glycerol). Cells were scored for protrusion on a scale of 1 (no protrusion) to 10 (maximum protrusion). Each condition was done in duplicate and scored by five different blinded observers who reported an average score across at least 10 image fields. Observer scores were averaged for each condition and then aggregated from different experiments.

### Rac1 and Cdc42 GTPase activity assays

Measurement of activated, GTP‐bound Rac1 and Cdc42 subfamily proteins was measured using a technique similar to the method described by Ren and colleagues [26]. OVCA433 cells transiently transfected with 5 ug of either pCMV‐Myc(J3)‐Cdc42‐63 L or pCMV‐Myc(J3)‐Rac1‐63 L expression plasmids, which produce constitutively active Myc‐Cdc42 or Myc‐Rac1 proteins, served as positive controls for the pulldown assay. Cells were lysed in 350 μL of 50 mm Tris, pH 7.4, 10 mm MgCl_2_, 500 mm NaCl, 1% Triton X‐100, 0.1% SDS, 0.5% deoxycholate, and protease inhibitors. Lysates (500–750 μg) were cleared at 16 000 × ***g*** for 5 min. Supernatants were rotated for 20 min with 30 μg GST‐PBD (GST fusion protein containing the Rac/Cdc42 binding domain of PAK1) bound to glutathione‐Sepharose beads (Cytiva, Marlborough, MA, USA). Samples were washed in 50 mm Tris, pH 7.4, 10 mm MgCl_2_, 150 mm NaCl, 1% Triton X‐100, and protease inhibitors. Pulldowns and lysates were then immunoblotted as described above for either GTPase (anti‐Cdc42 #2462; Cell Signaling Technology, Danvers, MA, USA or anti‐Rac1 #6106650; BD Transduction Labs, Franklin Lakes, NJ, USA).

### Rap1A/1B GTPase activation assays

Measurement of activated, GTP‐bound Rac1 and Cdc42 subfamily proteins was measured using a technique similar to the method described by Wittchen and colleagues [27]. Briefly, cells were lysed in 300 μL of 50 mm Tris, pH 7.4, 10 mm MgCl_2_, 75 mm NaCl, 1% Triton X‐100, 0.1% SDS, 0.5% deoxycholate, 1 mm sodium orthovanadate and protease inhibitors. 750 μg of lysates was cleared at 16 000 × ***g*** for 5 min, and the supernatant was rotated for 30 min with 50 μg GST‐RalGDS‐RBD bound to glutathione‐sepharose beads (Cytiva). Samples were washed in lysis buffer. GST‐RBD pulldowns and lysates were then immunoblotted as described above with an anti‐Rap1A/1B antibody (#2399; Cell Signaling Technology).

### Adhesion assays

24‐well plates were coated overnight (4 °C) with 10 μg cm^2^ Type I collagen (Rat Tail, Sigma Aldrich), Fibronectin (EMD Millipore), or Type IV collagen (Santa Cruz, CA, USA). Coated wells were blocked with a solution of 3% delipidated BSA (Sigma Aldrich)/PBS for 90 min at 37 °C, washed twice with 1 mL of PBS, and then air‐dried under sterile conditions. Control wells were not coated with a matrix before blocking with BSA/PBS. Cells were washed twice in PBS and detached from growth plates by incubating in Versene (Gibco) for 10 min. Collected cells were centrifuged, washed twice in serum‐free media, and resuspended to a concentration of 2 × 10^5^ per mL. One milliliter of cell suspension was placed in each well for the indicated time and unbound cells removed by washing with two 1 mL volumes of PBS. Adherent cells were fixed with 3.7% formaldehyde/PBS, stained with Crystal Violet (Carolina Biological, Burlington, NC, USA) for 10 min, and washed excessively with distilled water. Cell captured dye was solubilized with 0.5% SDS in water and the resulting solution's absorbance at 595 nm was assessed using a SpectraMax 190 plate reader. All conditions were done in triplicate, every plate contained its own matrix‐control wells, and background binding absorbance to control wells was subtracted from that of matrix coated wells. Normalized absorbance values for each condition were combined from multiple experiments.

### Statistical analysis

Membrane protrusion assay and cell adhesion data were analyzed by ANOVA. If significant, Tukey's range test of significance was utilized. Nonstimulated vs. EGF‐stimulated data adhesion data were analyzed by *t*‐test for each independent cell line.

## Results

**Fig. 1:**
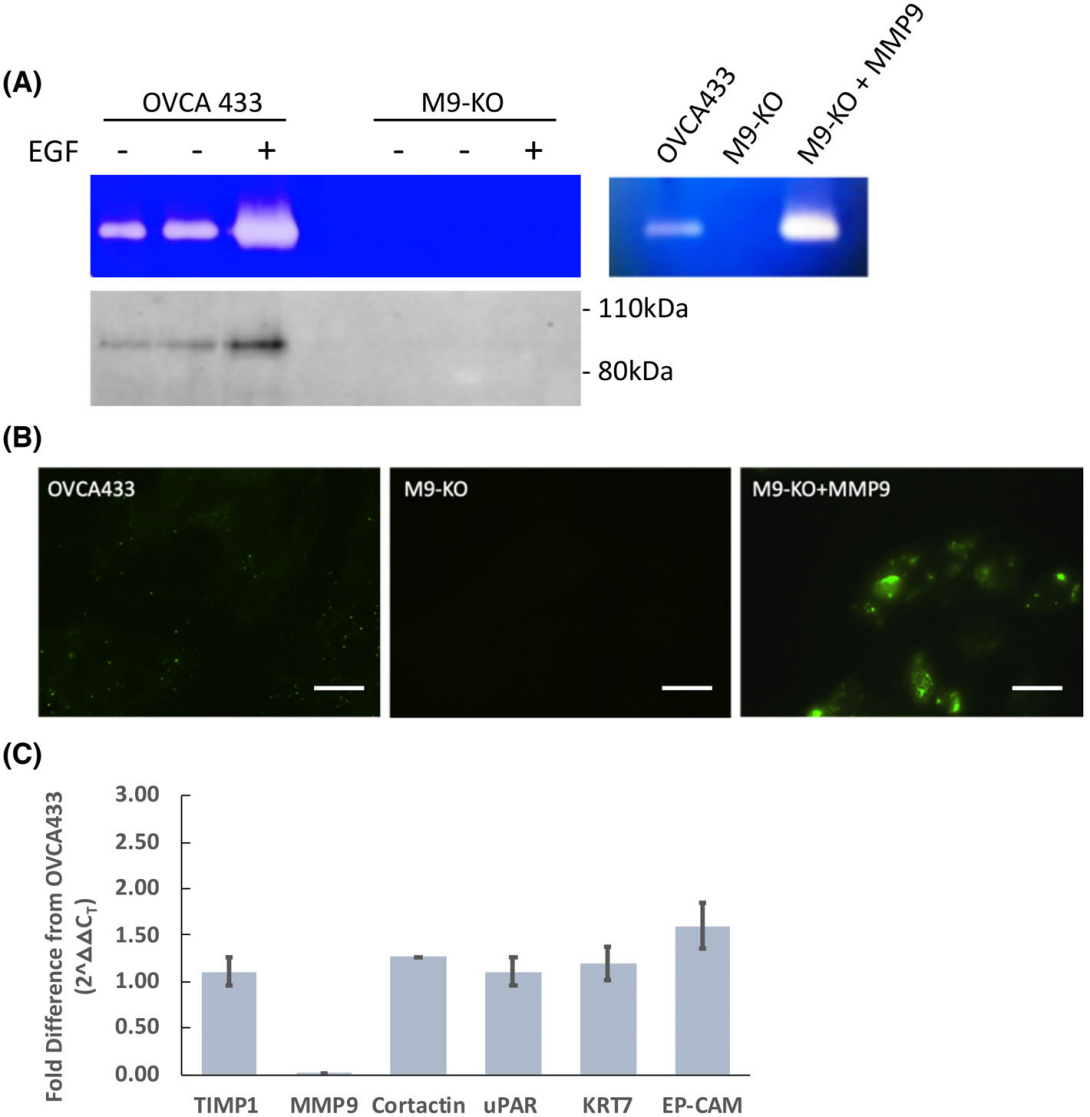
(A) Zymographic and western blot analysis of concentrated conditioned media confirm lack of MMP9 expression in OVCA433 cells with biallelic CRISPR‐mediated knockout of MMP9 (M9‐KO cells). As expected, EGF stimulated MMP9 release from OVCA433 cells. M9‐KO + MMP9 cells stably re‐express MMP‐9 lines at levels significantly higher than OVCA 433 cells. (B) Immunofluorescence of non‐permeabilized cells demonstrated punctate staining on the surface of OVCA433 cells, no detectable signal on M9‐KO cells, and substantial MMP‐9 signal in M9‐KO + MMP9 cells. Bar is 50 μm. Images represent three different experiments done in duplicate. (C) M9‐KO cells were confirmed via RT‐qPCR to lack detectable expression of MMP9 mRNA. M9‐KO same cells exhibited similar mRNA expression for TIMP‐1, cortactin, u‐PAR, Keratin7 (KRT7), and EP‐CAM as parental OVCA433 cells. Data represent three unique samples for each condition measured in triplicate and normalized to alpha‐Actin mRNA and OVCA433 expression for each transcript. Data are presented as fold expression difference +/− standard deviation.

MMP9‐null OVCA433 (M9‐KO) conditioned serum‐free media and cells were void of detectable MMP9 activity, protein, and mRNA (Fig. 1A–C). M9‐KO + MMP9 re‐expressing cells secreted noticeably higher amounts of MMP9 in conditioned media than OVCA433 cells (Fig. 1A). Expression of catalytically inactive MMP9‐H401A or MMP9‐H405A [28] in M9‐KO cells produced full‐length unreleased protein trapped in secretory vesicles (data not shown). MMP9 immunofluorescence staining of nonpermeabilized cells revealed heterogeneous punctate staining across OVCA433 cells, no detectable signal in M9‐KO cells, and significant MMP9 on the surface of M9‐KO + MMP9 cells (Fig. 1B). Analysis of M9‐KO cells confirmed the absence of MMP9 mRNA, while transcripts of TIMP1, cortactin, uPAR, KRT7, and EP‐CAM were all similar in expression levels compared to parental OVCA433 cells (Fig. 1C). Re‐expression of MMP9 in M9‐KO + MMP9 cells was accompanied by a 50% increase in endogenous TIMP1 mRNA expression (data not shown).

**Fig. 2:**
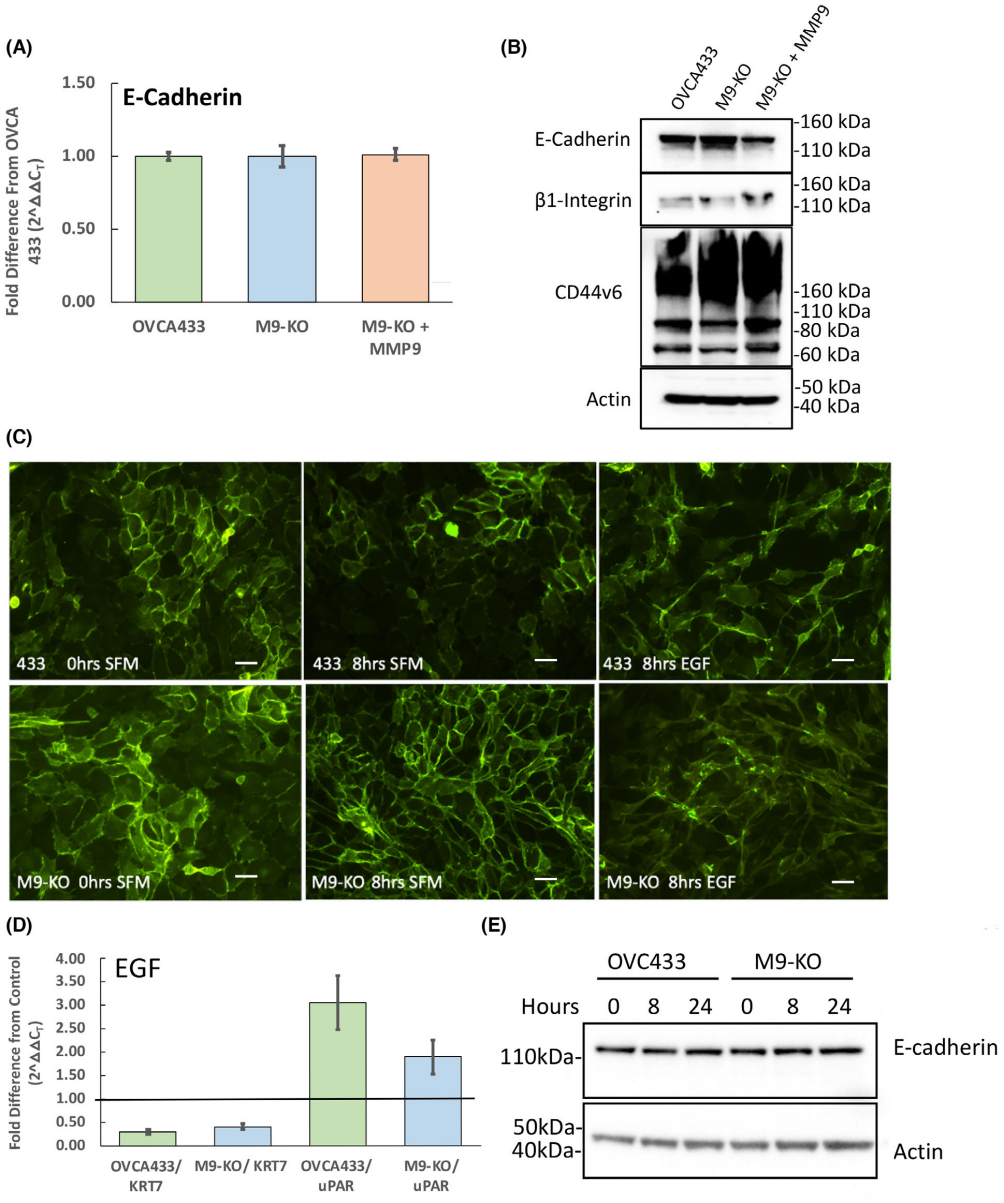
(A) Loss or gain of MMP9 had no impact on E‐cadherin mRNA expression as measured by RT‐qPCR. (B) At the same time, E‐cadherin protein expression was noticeably lower in lysates of M9‐K9 + MMP9 cells. There were no detectable differences in β1 integrin or CD44v6 protein expression. (C) EGF‐stimulated E‐cadherin junctional dissolution and produced a mesenchymal appearance in both OVCA433 and M9‐KO cells (8 h), with cells treated with serum‐free media (SFM) serving as a control. Bar is 50 μm. These data suggest MMP9 is dispensable for EGF‐driven epithelial to mesenchymal transition of OVCA433 cells. In support, a decrease in KRT7 and gain of uPAR mRNA expression was evident in both cell lines treated with EGF vs. control (D). (E) Analysis of E‐cadherin protein from whole cell lysates demonstrated the EGF‐stimulated EMT‐transition did not correlate with loss of E‐cadherin protein, even when examined after 24 h. Data for (A) and (D) represent three unique samples for each condition measured in triplicate and normalized to Actin mRNA and OVCA433 expression for each transcript. Data are presented as fold expression difference +/− standard deviation.

While all three cell lines had a similar level of E‐Cadherin mRNA (Fig. 2A), M9‐KO + MMP9 cells consistently displayed less E‐cadherin protein in cell lysates than the other cell lines (Fig. 2B). All three cell lines exhibited similar levels of beta one integrin and CD44v6 proteins (Fig. 2B). Both OVC433 and M9‐KO cell lines presented heterogeneous yet evident E‐cadherin junction staining (Fig. 2C, time 0 h). EGF‐stimulation reduced the amount of E‐cadherin border staining similarly in OVCA433 and M9‐KO cells within hours (Fig. 2C). This loss was accompanied by an epithelial‐to‐mesenchymal transition phenotype in both cell lines. Further, both cell lines exhibited loss of Keratin 7 and an increase in uPAR mRNA expression upon EGF‐treatment (Fig. 2D). Analysis of whole cell lysates revealed EGF‐stimulated E‐cadherin junctional dissolution was not accompanied by changes in E‐cadherin protein levels, even when analyzed 24 h after stimulation (Fig. 2E).

**Fig. 3:**
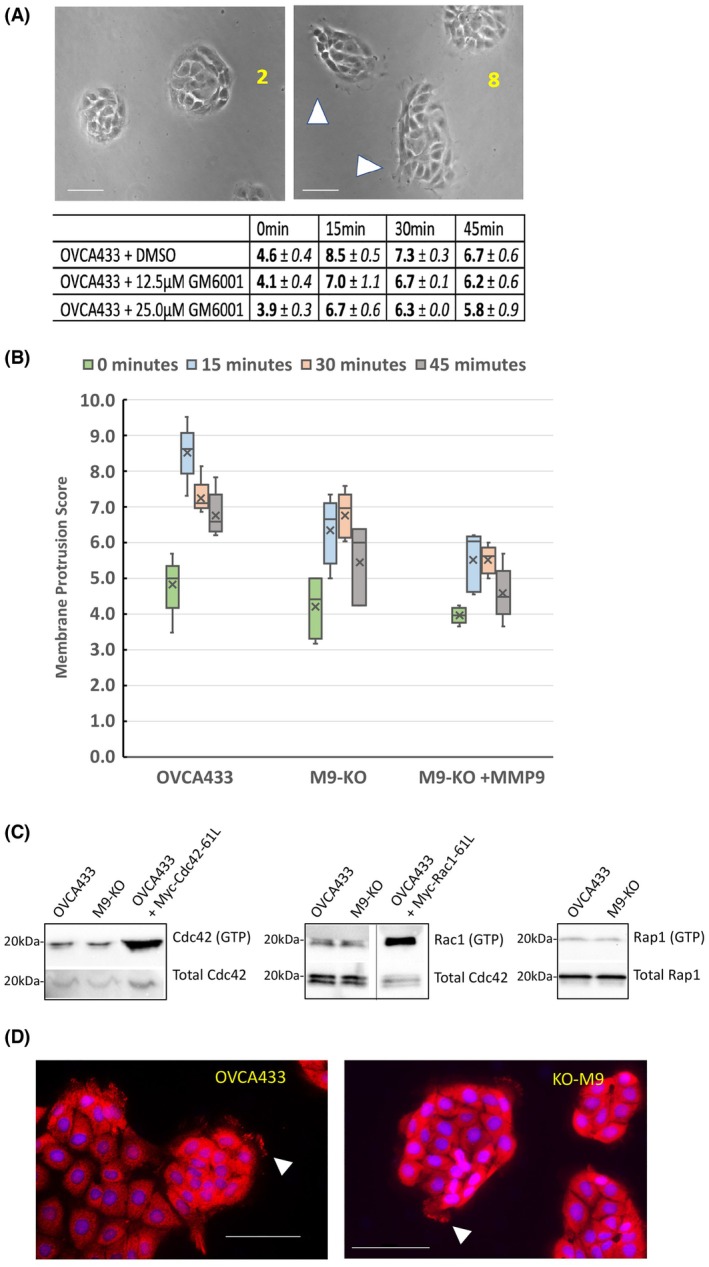
During phase‐contrast analysis, reduced membrane protrusion activity was noted in M9‐KO cells. We then scored all three cell lines for membrane protrusion activity (1 low, 10 high). (A) Examples of two scores (2 and 8) are provided. Bar is 100 μm. Note the amount of EGF‐driven membrane protrusions (arrowheads) for OVCA433 cells minutes after EGF stimulation. No differences in membrane protrusion were found in any cell line treated with control serum‐free media (*N* = 20, *P* = 0.89 for 0 min vs. 30 min serum‐free media). OVCA433 cells were stimulated with EGF in the presence of a DMSO vehicle or the indicated concentration of the MMP inhibitor GM6001. Bars represent standard deviations of normalized scores among all the observers. Data represent results from three unique experiments done in duplicate and presented with standard deviation. OVCA433 cell protrusion was maximal fifteen minutes after EGF stimulation before subsiding. Blocking extracellular MMP activity reduced the extent of EGF‐driven protrusion in a dose dependent fashion without changing the kinetics profile. As MMP9 is the primary extracellular MMP expressed by OVCA433 cells, these data suggest MMP9 activity directly contributes to membrane protrusion activity. ANOVA analysis of 0, 30, and 45 min samples showed no statistical significance. ANOVA analysis of 15 min samples were significant: F (2, 15)= 7.06 *P* = 0.0068 (OVCA433‐DMSO vs. OVCA433 12.5 μM GM6001 *P* = 0.026, OVCA433‐DMSO vs. OVCA433 25 μM GM6001 *P* = 0.00845) (B) OVCA433, M9‐KO, and M9‐KO + MMP9 cells were scored for EGF‐driven protrusion. Loss of MMP9 reduced the extent of EGF‐stimulated protrusions and produced a delay in maximal activity (15 vs. 30 min). Unexpectedly, stable re‐expression of MMP9 further reduced both basal (0 min) and EGF‐stimulated protrusion activity. Data are presented in box and whiskers format from five unique experiments performed in duplicate and scored by five blinded observers. X represents the mean and outer bars (whiskers) represent the variability outside the upper and lower quartile. ANOVA analysis of 0 min samples: F (2, 12)= 2.044 *P* = 0.1721 no statistical difference; ANOVA analysis of 15 min samples: F (2, 12)= 15.14 *P* = 0.00030 (OVCA433 vs. M9‐KO p = 0.0038, OVCA433 vs. M9‐KO + MMP9 *P* = 0.00029); ANOVA analysis of 30 min samples: F (2, 12)= 14.27 *P* = 0.00064 (OVCA433 vs. M9‐KO + MMP9 *P* = 0.00062, M9‐KO vs. M9‐KO + MMP9 *P* = 0.0074; ANOVA analysis of 45 min samples: F (2, 12)= 8.32 *P* = 0.0054 (OVCA433 vs. M9‐KO + MMP9 *P* = 0.0042). (C) OVCA433 and M9‐KO cells were analyzed for Rac1, Cdc42, and Rap1 activation (GTP bound). No detectable difference was found in GTPase activation state. Each blot represents at least three unique experiments. Exogenously expressed Myc‐Cdc42‐61 L and Myc‐Rac‐61 L served as positive control for GTP‐binding specificity. Myc‐Rac1‐61 L results were from the same sample blot but spaced apart to avoid signal contamination. These data suggest loss of MMP9 to did not disrupt the activation profile of key Rho family regulators even though lower membrane protrusion was evident in the absence of EGF (time 0). (D) β1‐integrin staining was evident in membrane projection in both OVCA433 and M9‐KO cells, indicating formation of β1‐integrin focal contacts was not disrupted, but delayed. Images represent two independent experiments completed in duplicate. Bar is 100 μm.

EGF stimulation of OVCA433 cells drove filopodia and lamellipodia protrusions within minutes (Fig. 3A). At the same time, preliminary phenotypic analysis by light microscopy and phalloidin staining of filamentous actin indicated loss of MMP9 reduces membrane protrusion activity (Fig. 3B). As this suggested involvement of MMP activity in membrane projections, OVCA 433 cells were treated with established MMP inhibitor GM6001. The addition of the MMP inhibitor significantly reduced both basal and EGF‐stimulated membrane protrusion (Fig. 3A, inset table). Time‐course analysis revealed EGF‐stimulation of OVCA 433 membrane protrusion was maximally detected at 15 min and was reduced by 30 min (Fig. 3A). In accordance with MMP inhibition data, M9‐KO cells exhibited delayed (maximum at 30 min) and reduced membrane protrusion compared to parent OVCA433 cells (Fig. 3B). The reduction in membrane protrusion in M9‐KO was appreciable before stimulation of EGF (Time 0), suggesting MMP9 contribution to membrane protrusion was not EGF‐specific. Cdc42, Rac1, and Rap1A GTPases have all been shown to govern membrane protrusive activity; however, under basal conditions, we found no differences between OVCA433 and M9‐KO cells in GTP‐binding, a reflection of their activation state (Fig. 3C). As there was no difference in CD44v6 and β1‐integrin expression in cell lysates (Fig. 2B), and the integrin protein was readily observable by immunofluorescence in membrane projections of both OVCA 433 and KO‐M9 cell lines (Fig. 3D), the mechanism behind reduced membrane protrusion (and adhesion) upon MMP9 loss did not correlate with a change in either expression of the protein. Unexpectedly, stable re‐expression of MMP9 further reduced both basal (0 min) and EGF‐stimulated protrusion activity (Fig. 3B).

**Fig. 4:**
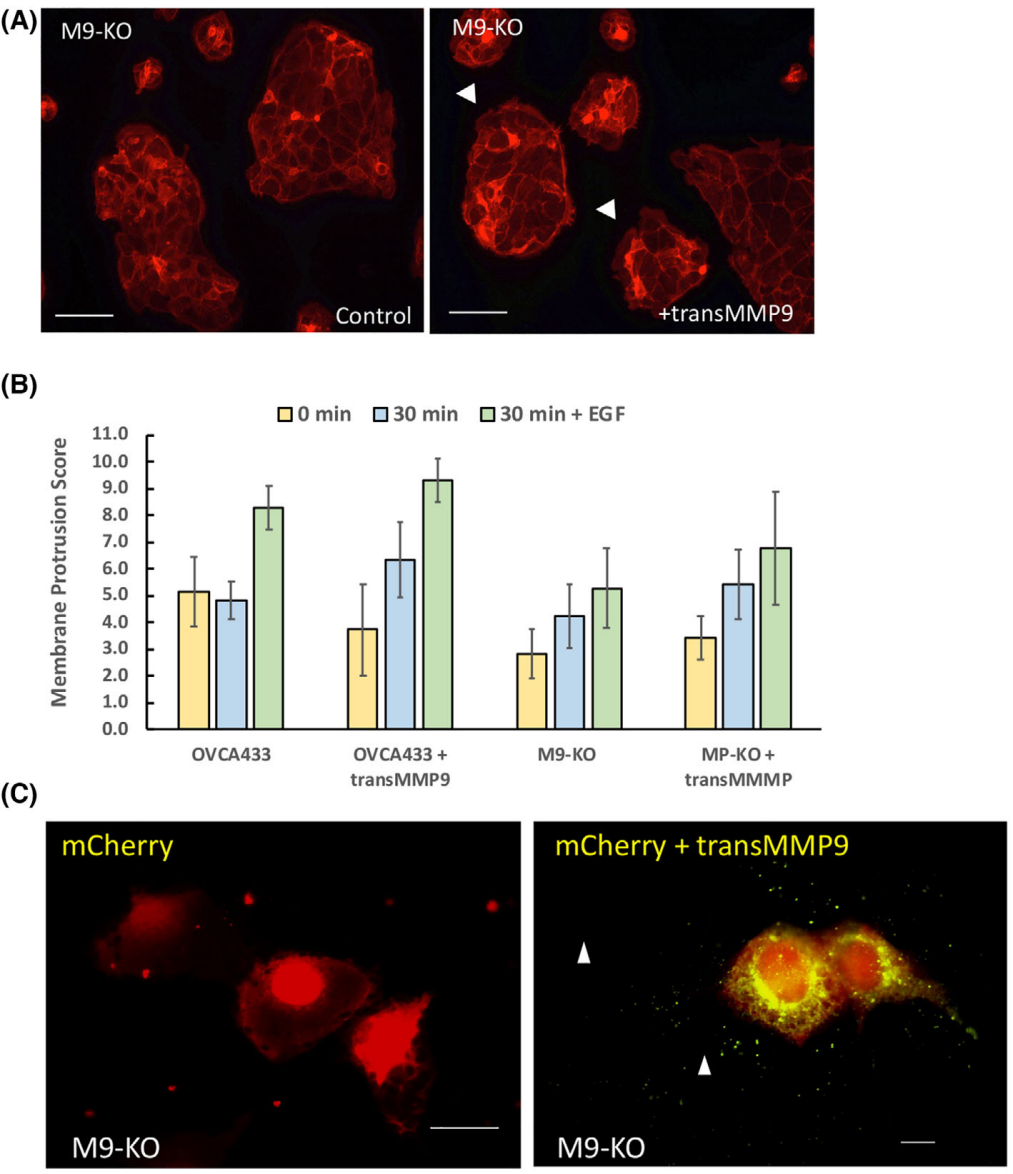
Transient expression of MMP9 stimulates membrane protrusion of M9‐KO cells. (A) Increased Actin‐based membrane protrusions were evident in populations of M9‐KO cells transiently transfected with pcDNA3.1‐MMP9 (+TransMMP9, see inset arrows) vs. control plasmid. Bar is 100 μm. (B) OVCA433 and M9‐KO cells were transiently transfected with control plasmid or pcDNA3.1‐MMP‐9 expression plasmid (+trans MMP9). Cells were scored for protrusion before (0 min) and after treatment with control serum‐free media (30 min) or EGF (30 min + EGF). Transient expression of MMP9 promoted an increase in membrane protrusion in both cell lines in the absence or presence of EGF. Data are from a represented experiment performed in duplicate, scored by blinded observers, and reported with +/− standard deviation. (C) M9‐KO cells were transfected with either pmCherry‐C1 alone or co‐transfected with pmCherry‐C1 and pcDNA3.1‐MMP9 expression plasmids together as indicated. As expected, MMP9 staining was not detected in M9‐KO cells transfected with pmCherry‐C1 alone. MMP‐9 was strongly expressed in co‐transfected cells, producing an overlapping yellow signal when merged with the red mCherry signal. Untransfected cells bound secreted MMP9 to their cell surface, producing a punctate signal that decreased as distance from transfected cells increased (arrows). These data provide evidence non‐expressing OVCA cells have access to a gradient of MMP‐9 released by neighboring cells. Bar = 25 μm (left) or 12.5 μm (right).

As M9‐KO + MMP9 cells produce more MMP9 than OVCA433 cells (Fig. 1A), we hypothesized that stable cells express too much of the enzyme for effective protrusion. To reduce the amount MMP9 re‐expressed, both OVCA433 and M9‐KO cells were transiently transfected with low amounts of pCDNA3.1‐MMP9 expression plasmid (< 5% expression efficiency by immunofluorescence, data not shown). Transient transfection of MMP‐9 produced a modest but detectable increase in membrane protrusions in both cell lines (Fig. 4A), even when the entire population of cells was examined by light microscopy (Fig. 4B). This increase was also evident in EGF‐stimulated cells. MMP9 staining of nonpermeabilized M9‐KO cells cotransfected with pCDNA3.1‐MMP9 and pmCherry‐C1 expression vectors revealed nonexpressing cells located near transfected ones bound extracellular MMP‐9 to their surface (Fig. 4C). MMP9 binding diminished as distance increased from expressing cells (Fig. 4C, arrowheads). These data provide evidence nonexpressing OVCA cells have access to a gradient of MMP‐9 released by neighboring cells.

**Fig. 5:**
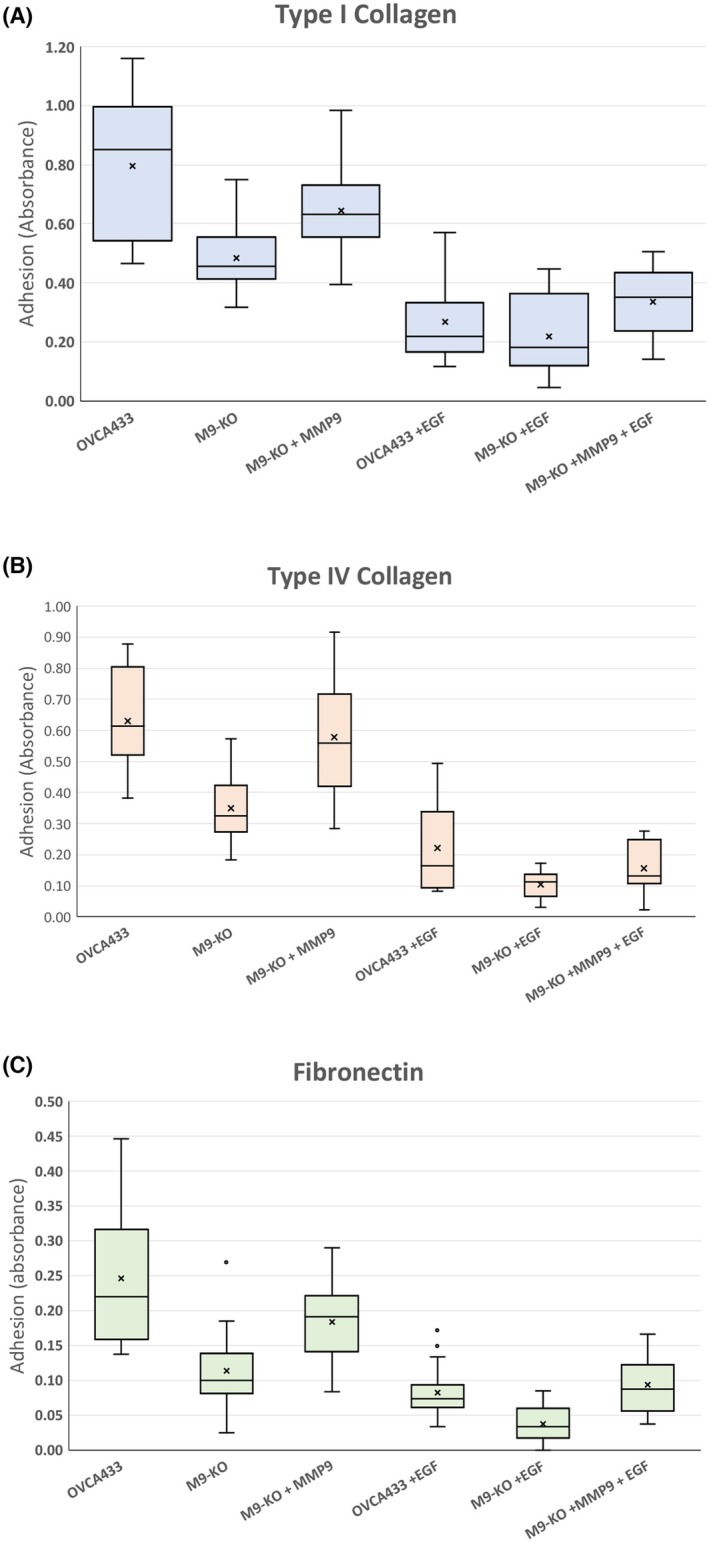
The indicated cells were adhered to matrix coated wells in the absence or presence of EGF as indicated. (A) For Type I Collagen, box and whisker values represent *N* = 18 samples from three different experiments. X represents the mean and outer bars (whiskers) represent the variability outside the upper and lower quartile. ANOVA analysis of non‐EGF treated samples: F (2, 49) = [14.66] *P* < 0.001. ANOVA analysis of EGF‐treated samples: F (2, 52) = [3.89] *P* = 0.02. Loss of MMP9 significantly reduced cell adhesion (p < 0.01 M9‐KO vs. OVCA 433). Re‐expression of MMP9 significantly recovered cell adhesion (*P* < 0.05 M9‐KO vs. M9‐KO + MMP9 cells). EGF significantly reduced cell adhesion for all three cell lines (*P* < 0.01). (B) For fibronectin, box and whisker values represent *N* = 18 samples from three different experiments. ANOVA analysis of non‐EGF treated samples: F (2, 50) = [15.55] *P* < 0.001. ANOVA analysis of EGF‐treated samples: F (2, 50) = [12.65] *P* < 0.001. Loss of MMP9 significantly reduced cell adhesion with and without EGF (*P* < 0.01 M9‐KO vs. OVCA 433). Re‐expression of MMP9 significantly recovered cell adhesion (*P* < 0.01 M9‐KO vs. M9‐KO + MMP9). EGF significantly reduced cell adhesion for all three cell lines (*P* < 0.01). (C) For Type IV Collagen, box and whisker values represent *N* = 12 samples from two different experiments. Open circles represent outlier data points that fall more than 1.5 times the interquartile range (IQR) away from the top or bottom of the box. ANOVA analysis of non‐EGF treated samples: F (2, 33) = 9.78 *P* = 0.000462. ANOVA analysis of EGF‐treated samples: F (2, 23) = 4.94 *P* = 0.0132. Loss of MMP9 significantly reduced cell adhesion (*P* < 0.01 M9‐KO vs. OVCA 433). Re‐expression of MMP9 significantly recovered cell adhesion (*P* < 0.01 M9‐KO vs. M9‐KO + MMP9 cells). OVCA 433 vs. M9‐KO + MMP9 cell adhesion was not significantly different. EGF significantly reduced cell adhesion for all three cell lines (*P* < 0.01). Together, these data indicate MMP9 directly promotes ovarian cancer cell adhesion to matrices common in mesothelium.

As MMP9 binds directly to and influences adhesion receptors [14, 15], we analyzed whether loss of MMP9 impacted OVCA 433 adhesion. Loss of MMP9 expression reduced OVCA433 adhesion to Collagen I (Fig. 5A), Collagen IV (Fig. 5B), and Fibronectin (Fig. 5C) matrices by 50–60%. Further, re‐expression of MMP9 in M9‐KO cells was sufficient to significantly restore adhesion to these matrices. Finally, we found EGF‐stimulation of suspended OVCA cells significantly reduced adhesion to all matrices examined, regardless of MMP9 expression status (Fig. 5A–C).

## Discussion

The most common metastasis of ovarian epithelial cancer is transcoelomic, possibly due to the physical proximity of the ovaries to the peritoneal cavity [29]. Ahmed et al. found addition of ascites fluid to the OVCA433 cell line used in our study increased their proliferation, adhesion, and invasion, suggesting that soluble factors in a patient's ascites fluid can promote transcoelomic metastasis [30]. Among growth factors and cytokine candidates, EGF stands out because of its concentration in malignant ascites fluid and the positive correlation between OEC cell EGFR expression and reduced patient survival [21, 22, 23, 24]. As EGF is an established promoter of MMP9 mRNA expression and protein secretion [22], we knocked out MMP9 gene expression in OVCA433 and identified consequences to EGF‐driven metastatic activity, specifically acquisition of an EMT phenotype, membrane protrusion, and cell adhesion.

We noted less E‐cadherin protein in whole cell lysates of M9‐KO stably re‐expressing MMP9 but E‐cadherin mRNA levels remained unchanged (Fig. 2). This suggests that MMP9, at sufficiently high enough concentration, can directly lead to turnover of E‐cadherin protein, likely by direct proteolysis. In support, Cowden Dahl et al. and others have reported that MMP9 cleaves E‐cadherin, possibly in promotion of its turnover following growth factor or lipid (e.g., lysophosphatidic acid) stimulation [1, 7, 31]. However, the ability of EGF‐stimulated M9‐KO cells to dissolute E‐cadherins and acquire an EMT phenotype in the absence of MMP9 expression provides clear evidence for the involvement of at least one alternative mechanism. As we did not observe loss of E‐cadherins protein in cell lysates upon EGF‐stimulation (Fig. 3E), we postulate E‐cadherin is being internalized and/or replaced with small and more dynamic adherens junctions, as previously proposed [32, 33].

EGF has previously been shown to drive membrane protrusion activity in a variety of cells, including epithelial, carcinoma, and fibroblast [34, 35, 36, 37, 38, 39]. Mechanisms behind EGF‐stimulated protrusion have largely been focused on intracellular events, especially small GTPase activity, subsequent actin severing, polymerization and branching, tension changes, and focal adhesion remodeling [34, 37, 39, 40, 41, 42]. While the work presented here is limited by genetic manipulation of just one cell line followed by two‐dimensional *in vitro* analysis, the findings provide strong evidence of a requisite role(s) for MMP9 in EGF‐stimulated membrane protrusion. Mechanistically, we found no detectable changes in Cdc42, Rac1, or Rap1A intracellular GTPase activity (Fig. 3C). Further, β1 and CD44 integrin expression, as well as β1 integrin staining, was evident in both cell lines (Figs 2C and 3D). While previous work has identified a noncatalytic binding role for MMP9 in the establishment of CD44 and β1 integrin based focal adhesion [43, 44], our results clearly identified MMP9 activity as necessary in the timing and strength of the membrane projection. It was initially surprising that, having established that transient expression of MMP9 in M9‐KO cells promoted modest membrane protrusions, M9‐KO cells that stably re‐expressed high amounts of MMP9 were even less protrusive than M9‐KO cells. We hypothesize that too little (none) or too much MMP9 activity impedes OVCA433 membrane protrusion. In support, we found untransfected cells were able to bind extracellular MMP9 released by neighboring transiently transfected ones, with cells in closest proximity binding MMP9 the strongest (Fig. 4C). This suggests that nonexpressing cells under transient transfection conditions have access to a gradient of MMP9 activity produced by neighboring transfected cells. Cell access to a gradient of MMP9 suitable for establishing and promoting membrane protrusions could mechanistically explain our otherwise contradictory results (Figs 3B and 4B).

EGF has been reported to stimulate loss of focal adhesions [45, 46, 47, 48]. Mukoyama et al. found EGFR activation stimulated β1 integrin internalization in squamous carcinoma cells, thereby reducing cell adhesion [49]. Ning et al. reported that EGF stimulates dissociation of EGFR and ɑ2 integrin in the OVCA433 cells used in our study, leading to integrin internalization within minutes [50]. Our work not only indicates that EGF reduces OEC cell adhesion but also does so independently of MMP9 expression status and when administered to suspended cells, such as those found in spheroids within ascites fluid.

We found that re‐expressing MMP9 in M9‐KO rescued loss of cellular adhesion to various matrices. Alford et al. found that MMP9 may act as a molecular scaffold for outside‐in signaling by dimerization through its hemopexin domain while simultaneously binding cell surface receptors EGFR alongside CD44 or ɑ4β1 integrin [44]. In support, the authors found inhibition of MMP9 dimerization and cell surface association through administration of an inhibitor to the enzyme's hemopexin domain was sufficient to reduce HT1080 cell adhesion to a mix of Type I collagen and Fibronectin. Our finding that MMP9 re‐expression could rescue cell adhesion but not membrane protrusion may reflect twin roles for MMP9 in cellular de‐adhesion through a window of permissive enzyme activity and adhesion through matrix receptor binding and trafficking. Future work will analyze surface changes in MMP9 complexes and binding partners in response to EGF stimulation. As TIMP1 has been postulated to have biological activity independent of its MMP inhibitory role [51, 52], we will also utilize the cell lines created and employed here to identify MMP9‐independent contributions of TIMP1 to ovarian cell metastatic activity.

## Conflict of interest

The authors declare no conflict of interest.

## Author contributions

SB, NE, NC, KM, CL, SR, and BK all contributed data, figures, and minor manuscript editing. CS and SE contributed data and created, wrote, and edited manuscript text and figures.

## Data Availability

The data that support the findings of this study are available from the corresponding author upon reasonable request.
